# An optimized isolation protocol yields high‐quality RNA from cassava tissues (*Manihot esculenta* Crantz)

**DOI:** 10.1002/2211-5463.12561

**Published:** 2019-02-20

**Authors:** Babak Behnam, Adriana Bohorquez‐Chaux, Oscar Fernando Castaneda‐Mendez, Hiroyuki Tsuji, Manabu Ishitani, Luis Augusto Becerra Lopez‐Lavalle

**Affiliations:** ^1^ Kihara Institute for Biological Research Yokohama City University Yokohama Japan; ^2^ International Center for Tropical Agriculture (CIAT) Valle del Cauca Colombia

**Keywords:** cassava, qRT‐PCR, RNA isolation, RNA sequencing

## Abstract

We developed and modified a precise, rapid, and reproducible protocol isolating high‐quality RNA from tissues of multiple varieties of cassava plants (*Manihot esculenta* Crantz). The resulting method is suitable for use in mini, midi, and maxi preparations and rapidly achieves high total RNA yields (170–600 μg·g^−1^) using low‐cost chemicals and consumables and with minimal contamination from polysaccharides, polyphenols, proteins, and other secondary metabolites. In particular, *A*_260_ : *A*_280_ ratios were > 2.0 for RNA from various tissues, and all of the present RNA samples yielded ribosomal integrity number values of greater than six. The resulting high purity and quality of isolated RNA will facilitate downstream applications (quantitative reverse transcriptase‐polymerase chain reaction or RNA sequencing) in cassava molecular breeding.

**Abbreviations**
cDNA: complementary DNA
qRT‐PCR: quantitative reverse transcriptase–polymerase chain reaction
RIN: ribosomal integrity number

Cassava (*Manihot esculenta* Crantz) is a tropical storage‐root crop that provides a staple source of food for over 800 million people globally 1. Therefore, cassava plants are a target crop for many molecular biology studies, including gene expression and transcriptome analyses of flowering for molecular breeding programs, and identification of transcripts that ameliorate biotic (virus and pests) and abiotic (drought and cold) stresses 1, 2, 3, 4, 5, 6, 7. Therefore, high‐quality RNA isolation from contaminants such as polysaccharides, polyphenols, proteins, and other secondary metabolites is crucial for molecular cloning, construction of cDNA libraries, and differential expression analyses of RNA‐Seq data using next‐generation sequencing.

Cassava (*M. esculenta*) belongs to the Euphorbiaceae family and contains various inhibitory compounds that strongly limit the extraction of high‐quality RNA from various cassava tissues 8. Several published studies demonstrate successful RNA extraction methods for various plant species under various environmental conditions 9, 10, 11, 12, 13, 14. Among these, classic total RNA extraction techniques involve single‐step extraction with acid guanidine thiocyanate/phenol/chloroform mixtures 15, and other RNA extraction protocols have been optimized for plants that are rich in phenols, polysaccharides, or other secondary metabolites, predominantly using sodium dodecyl sulfate (SDS), soluble polyvinylpyrrolidone (PVP), and ethanol precipitation 12. However, modified cetyltrimethylammonium bromide (CTAB) methods have been applied to specific plant species or tissues 9, 16, 17. In particular, Chang *et al*. 9 used a CTAB method to isolate RNA from pine tree tissues and achieved *A*_260_ : *A*_280_ ratios of only 1.7 to 2 and did not report *A*_260_ : *A*_230_ ratios. Similar to methods for species of the Euphorbiaceae family, such as *Manihot esculenta*,* Hevea brasilensis*,* Jatropha curcas*, and *Ricinus communis*, modified CTAB‐based protocols have been described as sources of better quality RNA. However, although *A*_260_ : *A*_280_ ratios were reportedly between 1.7 and 2.2, *A*_260_ : *A*_230_ ratios of 1.4 and −1.8 or less indicate sufficient contamination to preclude transcriptome analyses such as RNA sequencing 12, 18. Several reagents and kits are commercially available for isolating RNA from plants, including TRIZOL from Sigma‐Aldrich; RNeasy Plant Mini Kits from Qiagen; and RNAiso Plus from Takara. However, these kits fail in some plant tissues and in species with high polysaccharide, polyphenol, protein, and other secondary metabolite contents 12. Moreover, no standard RNA extraction method can be used to study tissues of cassava plants. Thus, we developed and modified a precise, rapid, and reproducible protocol that produces high total RNA yields with minimal contamination using low‐cost chemicals and consumables. Herein, we show that the present RNA isolation method produces high‐quality RNA from various tissues, including leaves, roots, and stems from young and mature cassava tissues. Furthermore, our high‐quality RNA isolation protocol is simple, non‐toxic, and produces sufficient quantities of high‐quality RNA.

## Materials and methods

### Plant materials

Multiple cassava tissues were collected from various genotypes of differing ages as follows:

#### Bioassays of cassava resistances to *Aleurotrachelus socialis* whitefl*y*

Several cassava genotypes were collected, and resistances to whitefly were compared with respective controls. Study genotypes including ECU72, COL1468, COL2246, TMS60444, PER368, PER317, and PER608 were obtained from *in vitro* culture systems and were propagated in the field to 8–10 weeks of age by planting in pots with sterile soil containing a 3 : 1 ratio of sand: black soil (no clay topsoil). Leaf tissues of various ages were infested with *A. socialis* and were then collected at various times until the last nymph stage of the insect. Samples were also collected from storage roots at times that corresponded with differential expression studies of beta‐carotene contents in roots of cassava strains at 12 months of age. Whole tissues from all stages of the experiment were immediately placed in liquid nitrogen and were stored at −80 °C.

#### Photoperiod studies of cassava tissues

The cassava genotypes HMC‐1 and GM3500‐2 (Esparrago) were studied as representatives of early and late flowering genotypes. Plants were propagated from stakes in the field and, after cleaning, were placed in hydroponic solution for 2 weeks 19, and were then transferred to pots. After 2 weeks of growth in greenhouse conditions, plants were transferred to growth chambers (Panasonic Incubator Serial MRL‐352) and were subjected to long daylight (LD, 929 lux) conditions (16 h light/8 h dark at 28 °C), or short daylight (SD, 929 lux) conditions (8 h light/16 h dark at 28 °C). After 3‐day acclimation, the youngest leaves, buds, and third and fourth leaves were sampled. Collected tissues from all stages of the experiment were placed immediately in liquid nitrogen and were stored at −80 °C.

#### Temperature treatments

To analyze the effects of temperature on cassava flowering times, HMC‐1 and Esparrago strains were grown at 15 °C and 30 °C under LD conditions *in vitro* and in pots (stakes from the field) using growth chambers (Panasonic Incubator Serial MRL‐352). Protocols for the preparation of stakes are detailed in part two. To prepare plants for *in vitro* experiments, tissue cultures were grown into plants and were then transferred to growth chambers for temperature treatments. After 3‐day acclimation, the youngest leaves, buds, and third and fourth leaves with their stems were sampled. Leaves, stems, and roots from *in vitro* plants were collected separately, and all collected tissues from all stages of the experiment were immediately placed in liquid nitrogen and stored at −80 °C.

### Solutions, reagents, and supplies

All stock reagents were purchased from commercial suppliers at appropriate stock concentrations, and only SDS and PVP40 were purchased as powders and were weighed and added directly to extraction buffers. The stock solutions UltraPure™ 1 m Tris/HCl (pH 7.5; Thermo Fisher Scientific), sodium chloride solution 5 m (Sigma‐Aldrich), UltraPure™ 0.5 m ethylenediaminetetraacetic acid (EDTA), (pH 8.0; Thermo Fisher Scientific), sodium dodecyl sulfate (SDS; Sigma‐Aldrich), polyvinyl pyrrolidone (PVP40; Sigma‐Aldrich), 2‐mercaptoethanol (Sigma‐Aldrich), UltraPure™ DNase/RNase‐free distilled water (Thermo Fisher Scientific), acid‐phenol: chloroform (pH 4.5) with isoamyl alcohol at a final ratio of 25 : 24 : 1 (Thermo Fisher Scientific), chloroform/isoamyl alcohol mixture (Sigma‐Aldrich), lithium chloride (8 m; Sigma‐Aldrich), ethanol absolute for analysis (Merck), chloroform for analysis (Merck), and RNaseZap™ RNase decontamination wipes (Invitrogen) were of molecular biology grade and were free of RNAses, DNAses, and pyrogens. All plastic supplies for the preparation of extraction buffer and the tubes used for extraction were disposable and were free of RNAses, DNAses, and pyrogens. We avoided the use of reagents with acute toxicity, such as diethyl pyrocarbonate, which is frequently used to inactivate RNAses.

### RNA extraction procedure

The following RNA isolation methods were used for all RNA preparation scales, including mini preparations of up to 2 μg of total RNA for reverse transcription‐polymerase chain reaction (RT‐PCR) analysis and midi or maxi preparations of 20 μg of total RNA for RNA‐Seq analyses. Prior to extracting RNA, benchtop areas and pipettes with filter tips were cleaned using RNAse zap wipes to inactivate RNAses. All procedures were performed using nitrile or latex gloves without powder.

### RNA extraction for mini and maxi preparations

Extraction buffer containing 100 mm Tris/HCl, 100 mm NaCl, 25 mm EDTA, 1% SDS, 2% PVP40, and 2% 2‐mercaptoethanol was prepared daily in ultra‐pure water that was free of RNase and was then preheated to 65 °C.

Tissues in liquid nitrogen were ground to a fine powder using a mortar and pestle for maxi preparations or a tissue lysis machine for mini‐scale preparations. Liquid nitrogen was continuously provided to avoid melting of frozen tissues and to prevent RNA degradation.

Subsequently, for mini‐scale preparations, 100 mg samples of powdered tissues were added to 2.0‐mL tubes under liquid nitrogen, and 700 μL aliquots of preheated extraction buffer were then added and vortexed. After adding and vortexing of 700 μL aliquots of chloroform, mixtures were centrifuged at 15 000 ***g*** for 20 min at 4 °C, and supernatants were transferred to fresh 2‐mL tubes and were washed with chloroform two more times by shaking and inverting. Supernatants were then transferred to 2‐mL tubes with care not to contaminate with lower chloroform phases, and RNA was precipitated from supernatants (1.5 mL) by adding 0.33 volumes (0.5 mL) of 8 m lithium chloride and incubating at 4 °C overnight.

On the following day, mixtures were centrifuged at 15 000 ***g*** for 10 min at 4 °C, supernatants were discarded, and precipitates were dissolved in 500 μL of ultra‐pure water. Poor aqueous solubility of precipitates at this stage is indicative of high RNA quality. Subsequently, RNA was extracted by centrifuging at 15 000 ***g*** for 10 min at 4 °C in one volume of acidic phenol: chloroform: isoamyl alcohol (25 : 24 : 1) and then in one volume of chloroform: isoamyl alcohol (24 : 1). Supernatants (500 μL) were then transferred to fresh tubes, and RNA was precipitated using 0.25 volumes of 5 M NaCl (125 μL) and 2.5 volumes of ice‐cold (−20 °C) absolute ethanol (1250 μL) for ≥30 min at −80 °C. Mixtures were then centrifuged at 15 000 ***g*** for 20 min at 4 °C. After discarding supernatants, precipitated pellets were washed immediately with 70% ethanol and centrifuged again at 15 000 ***g*** for 10 min at 4 °C. Finally, supernatants were discarded, and RNA pellets were air‐dried for 5–20 min and then resuspended in 20–100 μL of ultra‐pure RNAse free water, depending on yield and preparation scale, and were stored at −80 °C.

Application of the above protocol for midi or maxi scale requires proportional scaling of sample, initial extraction buffer, chloroform volumes, and centrifuge speeds (Table 1).

**Table 1:** Volumes and spin speeds for the first day of the extraction protocol are presented according to scales of RNA preparations

	Miniprep	Midiprep	Maxiprep
Initial amount of tissue	100 mg	1 g	3 g
Buffer and chloroform volume per sample	700 μL	5 mL	14 mL
Spin speed	15 000 ***g***	10 000 ***g***	8500 ***g***

**Figure 1:**
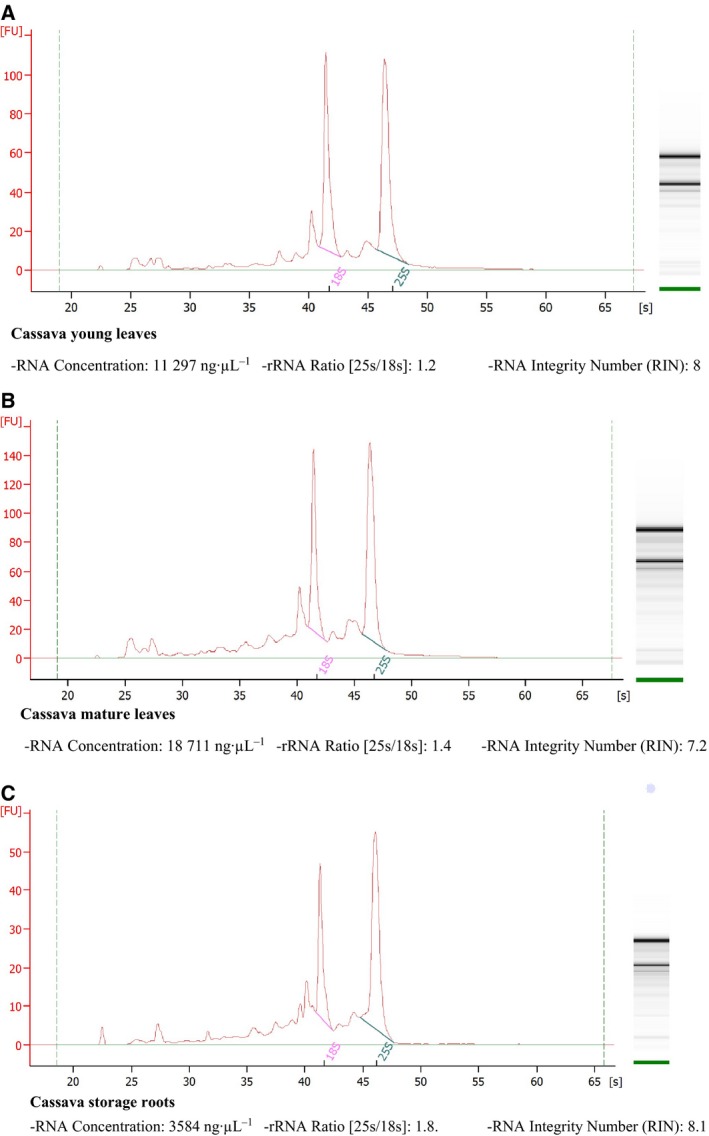
Electropherograms of total RNA from cassava obtained using our method showing 18S and 25S rRNA regions with RNA concentrations and RIN values; (A) to (C) correspond with RNA from leaves and storage roots, and (A) and (B) correspond with RNA from different stages of plant development (young and mature leaves). RNA were visualized in denaturing agarose gels stained with SYBR safe. RNA were analyzed using Agilent RNA 6000 Nano Assays in a 2100 Bioanalyzer (Agilent Technologies) and were then used for RNA sequencing.

Quantities and qualities of isolated RNA were evaluated spectrophotometrically by determining absorbance ratios of *A*_260_ : *A*_280_ and *A*_260_ : *A*_230_ using a Nanodrop^®^ ND‐1000 spectrophotometer (Thermo Fisher Scientific). RNA quality was further assessed electrophoretically on 1% denaturing agarose gels, which were prepared by adding 0.5 g of agar powder to 50 mL of ultra‐pure RNase free water and boiling until melted, followed by the addition of 8.75 mL of formaldehyde and 5 mL of 10 × MOPS buffer. Gels were run at 90 V for about 45 min. RNA integrity was assessed using an Agilent RNA 6000 Nano Kit with an Agilent 2100 Bioanalyzer (Agilent Technologies; www.agilent.com/genomics/bioanalyzer; Figs 1 and S3).

### RT‐PCR and qRT‐PCR

Total RNA (2 μg aliquots) were isolated from tissues of HMC‐1 and Esparrago (ESP) cassava genotypes and were treated with 1 U of DNase I (Promega). First‐strand cDNA were then synthesized in reaction mixtures containing total RNA, anchored oligo‐(dT)20 primer, and SuperScript™ III Reverse Transcriptase (Invitrogen) as described in the manufacturer's instructions. To confirm the absence of DNA contamination, a 412‐bp region of the *G3pdh* gene was amplified using PCRs with the primers GDPX7F (GATAGATTTGGAATTGTTGAGG) and GDPX9R (AAGCAATTCCAGCCTTGG). PCR mixtures were initially denatured at 94 °C for 5 min and were then subjected to 36 cycles of 94 °C for 30 s, 55 °C for 30 s, and 72 °C for 20 s, and a final extension of 72 °C for 5 min. Amplified products were then separated on 1% agarose gels and were stained with SYBR™ Safe DNA Gel Stain (Invitrogen). Finally, 75 ng cDNA specimens were subjected to quantitative reverse transcriptase‐polymerase chain reactions (qRT‐PCRs) in triplicate using Brilliant II SYBR Green PCR kits (Agilent Technologies) as described in the Rotor‐Gene Q (Qiagen) protocol with the primers 18S‐Fw (ATGATAACTCGACGGATCGC) and 18S‐Rv (CTTGGATGTGGTAGCCGTTT), which amplified a 169‐bp 3ʹ sequence of the 18S gene. A reverse transcription non‐template negative control was included to confirm the absence of genomic DNA, primer‐dimers, and other unfavorable reactions in RT‐PCR and qRT‐PCR analyses.

## Results and Discussion

Initially, we extracted RNA from cassava leaves using TRIZOL reagent according to previously described methods 20. However, the resulting RNA was of low yield and purity, and subsequent application of high‐salt solution failed to improve RNA quality and yield (Table S1). These experiments indicate that TRIZOL‐based methods are not suitable for cassava tissues, likely reflecting the prevalence of inhibitory compounds in members of the Euphorbiaceae family, such as cassava (*M. esculenta*).

Previous studies show that polyphenolic compounds oxidize and covalently link with quinones, which bind to RNA and form high molecular weight complexes 21. Moreover, polysaccharides reportedly coprecipitate with RNA in the presence of alcohols and remain as contaminants in final steps of RNA extraction, thus hampering subsequent molecular applications 8. Therefore, to remove polyphenols, polysaccharides, proteins, and other unnecessary secondary metabolites of isolated RNA more efficiently, we modified the methods reported by Chang *et al*. 9, by removing CTAB and spermidine from the extraction buffer and adding SDS (1%) and using several additional chloroform washing steps (three on the first day) and single washes with acidic phenol: chloroform: isoamyl alcohol (25 : 24 : 1) and chloroform: isoamyl alcohol (24 : 1, on the second day). In subsequent spectrometric assessments, *A*_260_ : *A*_280_ ratios increased to 2.02–2.2 indicating very low protein contamination, and *A*_260_ : *A*_230_ ratios of 2.03–2.29, except in one sample (1.93; Table 2 and Table S2), indicated high nucleic acid purity. In addition, the extraction protocol described here efficiently yielded 170–600 μg·g^−1^ of high‐quality total RNA from all types of cassava tissues under all treatment conditions (Table 2 and Table S2). These yields were very high in comparison with all other methods 8, 9, 10, 11, 12.

**Table 2:** Yields and *A*_260_ : *A*_280_ and *A*_260_ : *A*_230_ ratios of isolated total RNA from various cassava tissues

Name	Tissue	Yield (ng·μL^−1^)	260/280	260/230
85	HMC‐1 0H‐LD, Third leaf	2733.7	1.97	2.03
86	HMC‐1 4H‐LD, Third leaf	1921.1	2.02	2.07
87	HMC‐1 8H‐LD, Third leaf	1694.1	2.08	2.23
88	HMC‐1 12H‐LD, Third leaf	1483.2	2.08	2.14
89	HMC‐1 16H‐LD, Third leaf	2181.2	2.05	2.09
90	HMC‐1 20H‐LD, Third leaf	2469.2	2.05	2.17
91	HMC‐1 24H‐LD, Third leaf	2565.6	2.06	2.23
197	Esparrago 0H‐LD, Third leaf	2734.5	2.04	2.14
198	Esparrago 4H‐LD, Third leaf	2699.5	2.03	2,11
199	Esparrago 8H‐LD, Third leaf	2321.4	2.01	2.08
200	Esparrago 12H‐LD, Third leaf	2320.9	2.05	2.15
201	Esparrago 16H‐LD, Third leaf	2431.2	2.04	2.28
202	Esparrago 20H‐LD, Third leaf	2605.4	1.99	2.21
203	Esparrago 24H‐LD, Third leaf	2272.2	2.03	2.28
TL48	HMC‐1 Buds and young leaves Rep I (from Pot in 15 °C)	861.7	2.08	2.2
TL54	HMC‐1 Stem Rep I (from Pot in 15 °C)	1062.4	2.14	2.07
IL126	HMC‐1 Roots from RepI (from *in vitro* samples in 30 °C)	842.8	2.1	1.93
IL138	HMC‐1 Leaves RepII (from *in vitro* samples in 30 °C)	1994.1	2.1	2.22
IL141	HMC‐1 Stem from RepII (from *in vitro* samples in 30 °C)	1454.5	2.1	2.12
IL144	HMC‐1 Roots from RepII (from *in vitro* samples in 30 °C)	605.8	2.08	1.81
ECU 72‐1	ECU 72‐1 Cassava storage roots^a^	245	2.06	2.3
ECU 72‐2	ECU 72‐2 Cassava young leaves^a^	713	2.1	2.11
ECU 72‐3	ECU 72‐3 Cassava mature leaves^a^	402	2	2.01

a RNA mid‐preparation scale, the other ones were isolated with mini‐preparation scale.

Although some commercial kits, such as RNaesy Plant Mini Kit (Qiagen) and Pure link RNA Reagent (Thermo Fisher), are available for RNA extraction, these kits are not designed for analyzing plant tissues containing high starch concentrations, such as cassava plant tissues. The use of these kits without any additional purification steps for such samples dramatically reduces the quality and quantity of extracted RNA. Our past experience with the RNaesy Plant Mini Kit revealed its applicability only for fresh leaf samples or young tissues and not for old leaf tissues or cassava leaves, which are used for treating conditions such as infestation and other stresses, owing to a dramatically reduced quantity and quality of extracted RNA (Table S1).

Furthermore, it is obvious that research cost is an extremely critical aspect of a research project. Most of these kits are expensive, and the cost drastically increases with an increased number of samples to be analyzed or with a high concentration of high‐quality RNA requirement. Table S3 depicts the comparison of the cost incurred to obtain 1‐μg RNA using these kits and using our methods, revealing that our method is significantly cost‐effective. For instance, the cost for extracting 1‐μg, high‐quality RNA by our method is approximately 0.23 USD and that for extracting 1‐μg, high‐quality RNA by the RNaesy Plant Mini Kit is 7.62 USD, STRN50‐1 Kit (Sigma) is 0.98 USD, and PicoPure RNA Isolation Kit (Life Science Technologies) is 0.48 USD.

Moreover, some of these kits require specific conditions for certain plant tissues, whereas our method is suitable for various tissues of differing ages under different experimental conditions**.** Utsumi *et al*. 22 used the RNaesy Plant Mini Kit for RNA extraction from cassava leaves for transcriptome analysis (microarray) using an oligonucleotide DNA microarray in response to an infection by the fungus *Colletotrichum gloeosporioides*. However, there is no information regarding the efficiency of the RNaesy Plant Mini Kit for RNA extraction from cassava plants. Wilson *et al*. 23 used the Plant Total RNA 88 Kit (Sigma) for RNA extraction from non‐meristematic cassava tissues and the Arcturus PicoPure 89 RNA Isolation Kit for total RNA extraction from the shoot and root apical meristematic (SAM and RAM, respectively) cassava tissues. However, these kits are expensive and suitable only for analyzing a small amount of RNA from SAM and RAM, which reduces their applicability for RNA‐Seq library construction and qRT‐PCR, which require a large amount of RNA. Bowrin *et al*. 24 employed a formamide‐based methodology for RNA isolation only from tuber tissues of the cassava plant. Tuber tissue cores were blended in 100% formamide to make a smooth puree that was stored at 4 °C for 1, 4, and 7 days to test for the effect of storage on RNA integrity. Although the authors simplified the method by eliminating the use of liquid nitrogen or lyophilization, the A_260_/A_280_ ratio was not 2, the 26S rRNA concentration (except on day 1) was low, and no information was obtained regarding the RIN value and the yield of the extracted RNA. In addition, our method has demonstrated applicability not only for tuber tissues but also for other tissues such as leaf and stem.

Although the extraction buffer used here contained SDS, Tris/HCl, EDTA, sodium chloride, PVP, and β‐mercaptoethanol, as in former protocols, we determined optimal concentrations of these constituents for Euphorbiaceae family plants and confirmed that 2% SDS, 2% PVP, and 1–2% β‐mercaptoethanol (Table 2 and Table S2) produce the highest RNA yields 9, 12. SDS is a strong anionic detergent that can solubilize lipid proteins and lipids and is widely used to disrupt cells for RNA isolation. Moreover, β‐mercaptoethanol is widely used to inhibit RNase activity and prevent sample oxidation. Numerous previous studies show the efficacy of sodium chloride during extraction of nucleic acids from polysaccharides 9, 10, 12, 25, 26, 27, and although sodium chloride decreased RNA yields in some studies 10, 12, we achieved high RNA yields in its presence. These observations suggest that PVP and EDTA increased the present RNA yields, and in agreement with a previous study 10, we confirmed that addition of PVP powder to extraction buffer contributed to these improvements. We also performed multiple chloroform washes to remove debris and proteins in the initial stages of RNA isolation, and the use of 8 M lithium chloride likely facilitated RNA precipitation. Phenol was also used as an alternative to TRIZOL reagent and gave better results with lower costs. Former studies show that phenol is more suitable for high‐throughput RNA extraction from a broad range of plants in developmental stages. Moreover, acidic phenol lowers the pH and decreases DNA contamination 10, and phenol: chloroform and chloroform: isoamyl alcohol solutions promoted protein precipitation and decreased polysaccharide contents 28. Moreover, in accordance with previous comparisons by Chang *et al*. 1993, we added high concentrations of lithium chloride to samples prior to overnight incubation after the first day to limit DNA contamination 8, 9, 29. During the final stages of our RNA extraction protocol, 100% ethanol and 5 M sodium chloride were used to precipitate pure RNA. In subsequent analyses using an Agilent Bioanalyzer 2100, we determined the quality of RNA from various cassava tissues and showed ribosomal integrity numbers (RINs) of > 6 (Fig. 1). These data indicate suitability of our RNA isolates for high stringency applications such as cDNA library construction and RNA sequencing and for > 6 months storage, as shown in a previous study 30.

As it has been mentioned, in analyses of purity, *A*_260_ : *A*_280_ ratios of our RNA from various cassava tissues and varieties were all > 2.0, indicating high purity of isolated RNA. Although this ratio is an important indicator of sample quality, the best indicator of RNA integrity is functionality in subsequent applications. Thus, we evaluated RNA integrity according to RIN using a 2100 Bioanalyzer instrument with Agilent RNA 6000 Nano Assay kits (Agilent Technologies). These analyses showed high RIN values in all cases (6 to 8.1; Fig. 1), indicating excellent RNA quality of total RNA from young cassava leaves (Fig. 1A), mature cassava leaves (Fig. 1B), and roots (Figs 1C and S3), and effective removal of polyphenol and polysaccharide contents of these tissues.

**Figure 2:**
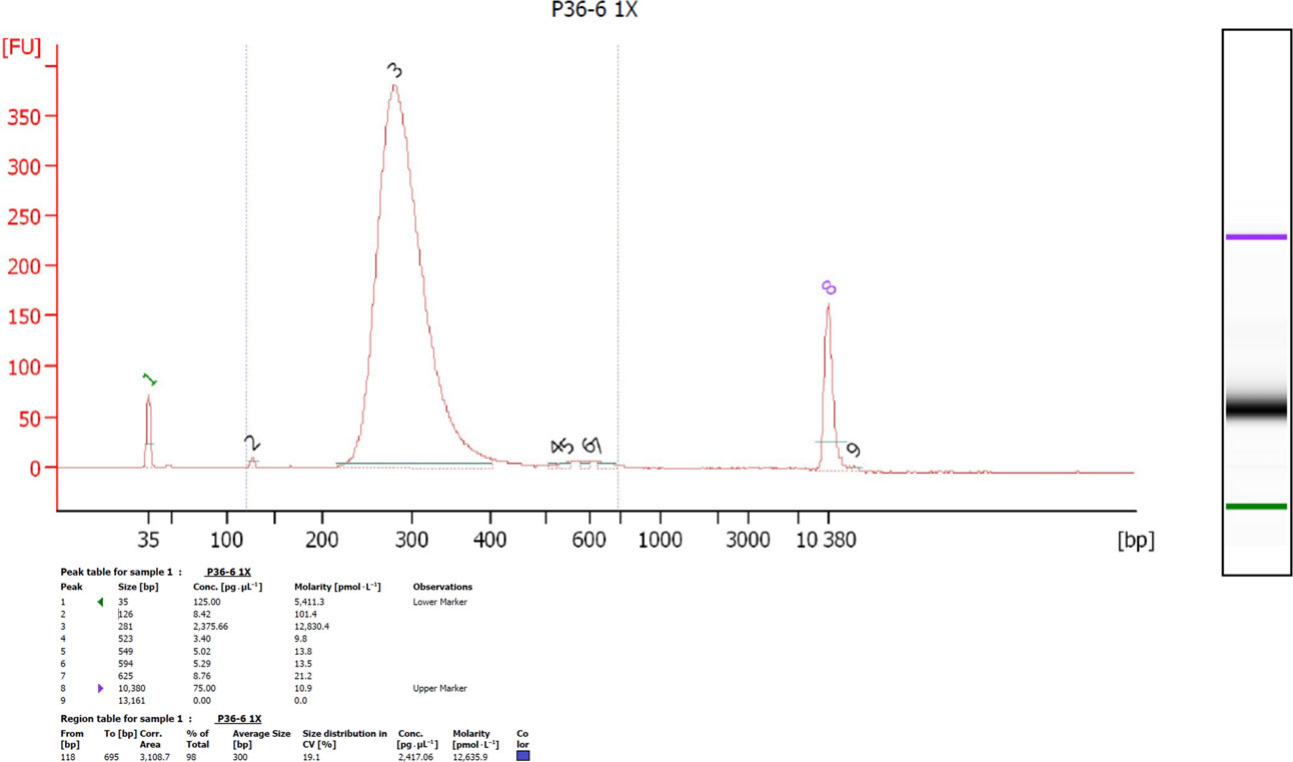
Electropherogram of sequencing libraries; the graph shows length distribution curves of sequencing libraries obtained using a low‐cost library construction protocol 18. Curves were generated on a 2100 Bioanalyzer using a DNA 1000 chip (Agilent Technologies). The photograph was provided by Maria Irigoyen and Linda Walling, University of California, Riverside.

Some studies have reported next‐generation sequencing or RNA library construction using RNA samples with RIN values of 5.5–6.5. Sánchez *et al*. 31 performed RNA extraction for Maqui berry samples according to the adapted CTAB method using high concentrations of PVP (4%), β‐mercaptoethanol (4%), and spermidine in the extraction buffer. All the samples provided RIN values of > 5, representing good RNA quality for downstream applications (i.e., next‐generation sequencing analysis). Chaudhary *et al*. 32 used the Invitrogen Kit (pure Link RNA Micro kit; cat. no. 12183‐016) for total RNA extraction. The high‐quality, extracted RNA had a RIN value of > 6, whereas our extracted RNA in several cases had a RIN value of > 6.6. For instance, in this study, case no. 1 (see plant material in the Materials and Methods section), the total number of samples of extracted RNA was 306 with a high yield (> 6 μg/1 mg tissue) and high quality by RIN value of 6.6–8.1 (Fig. 1). In addition, we randomly evaluated some of the extracted RNA samples from photoperiod experiments and obtained a RIN value of > 9 (Fig. S4); these samples were successfully used in downstream applications for constructing RNA library. Our collaborator, Linda Walling at the University of California, Riverside, had made over 270 libraries from our extracted RNA. They have sequenced libraries on the Illumina HiSeq2500 or NextSeq500 with 8 and 12‐fold multiplexing, respectively. There was no failed in the libraries due to the high quality of the RNA. Transcriptomics analyses using next‐generation sequencing require high concentrations and qualities of clean RNA material. There was no failed in the libraries due to the high quality of the RNA for the construction of cassava RNA libraries for all studies of whitefly resistance. Specifically, we sent total RNA to Linda Walling’s laboratory at the University of California, Riverside, and had libraries built using the low‐cost library construction protocols designed by Wang *et al*. 18 (Figs 2 and S5).

**Figure 3:**
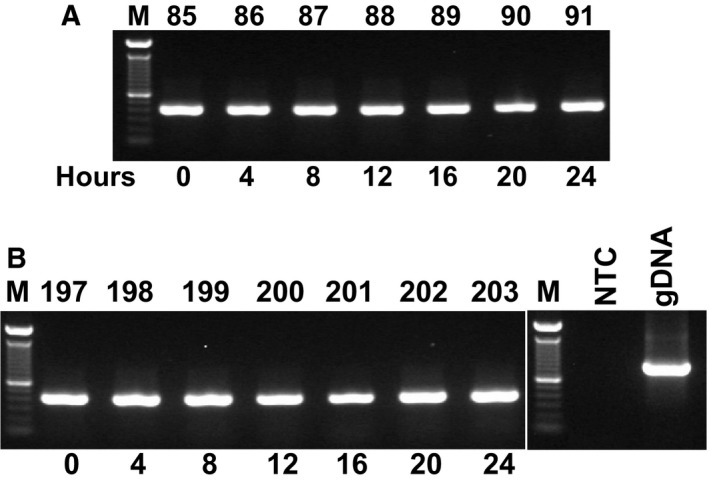
Conventional PCR with G3pdh primers was performed using cDNA that was reverse‐transcribed from total RNA after extraction using the present protocol. Tissue samples were taken from HMC‐1 (A) and Esparrago (ESP; (B) genotypes (Manihot esculenta) that were grown under long‐day (LD) conditions for indicated times; M: 1 Kb ladder; 85: HMC‐1, 0 h LD; 86: HMC‐1, 4 h LD; 87: HMC‐1, 8 h LD; 88: HMC‐1, 12 h LD; 89: HMC‐1, 16 h LD; 90: HMC‐1, 20 h LD; 91: HMC‐1, 24 h LD; 197: ESP, 0 h LD; 198: ESP, 4 h LD; 199: ESP, 8 h LD; 200: ESP, 12 h LD; 201: ESP, 16 h LD; 202: ESP, 20 h LD; 203: ESP, 24 h LD; NTC: non‐template negative control (water template), and gDNA: cassava genomic DNA.

**Figure 4:**
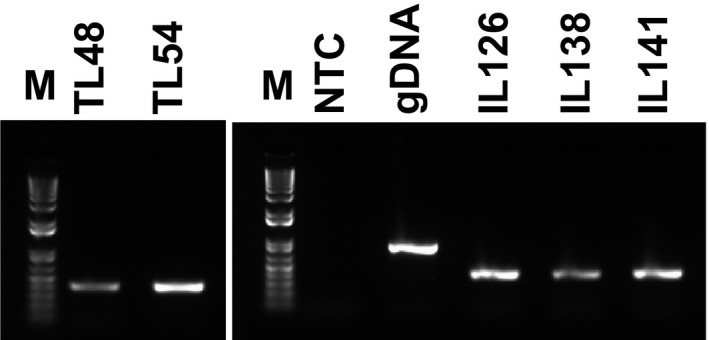
Conventional PCR using primers for G3pdh and cDNA from total RNA that was extracted from various tissues of the ESP genotype grown in pots and *in vitro* at 15 °C and 30 °C under LD conditions for 16 h inside growth chambers. Biological repeat 1: left panel; M, 1 Kb ladder; TL48, buds and young leaves from potted plants at 15 °C; TL54, stems from potted plants grown at 15 °C; right panel, NTC, negative control, water template; gDNA, cassava genomic DNA; IL126, roots from *in vitro* samples grown at 30 °C; IL138, leaves from *in vitro* samples grown at 30 °C; biological repeat 2: IL141, stems from *in vitro* samples grown at 30 °C. See Fig. S2 for complete PCR analyses of total RNA from potted and *in vitro* samples.

Subsequently, to demonstrate functional intactness of isolated mRNA, we performed RT‐PCR analysis using RNA samples from cassava tissues. In these experiments, a 412‐bp *G3pdh* sequence was successfully amplified from cDNA, and the absence of the 865‐bp genomic DNA amplicon indicated no contamination with genomic DNA (Figs 3 and 4, Figs S1 and S2).

Moreover, these analyses were equally successful using RNA isolates from several types of cassava tissues and plants grown in pots or *in vitro*. These experiments confirm that the present method for isolating RNA is suitable for PCR amplification and results in RNA samples that are free of inhibitors. In agreement, it is well known that reverse transcriptase is highly sensitive to impurities 10, further confirming the quality of the present RNA isolates.

**Figure 5:**
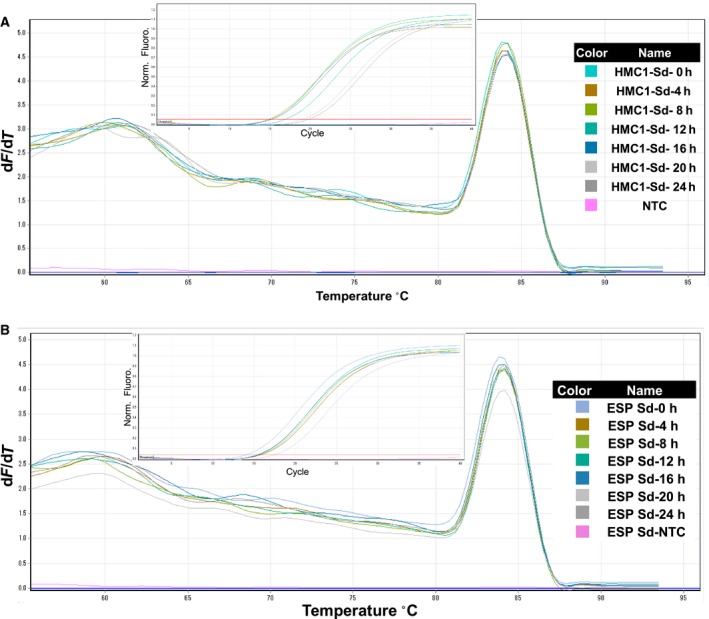
Quality analyses of cDNA from total RNA that was extracted using the present modified protocol; leaf samples were taken from the cassava genotypes HMC‐1 and ESP at indicated time points, and qRT‐PCR analyses were performed using primers for 18S. (A) Amplification plot and melting curves for HMC‐1 genotype; (B) amplification plot and melting curve for ESP genotype. For details of all curves, see Fig. 3 legend.

**Figure 6:**
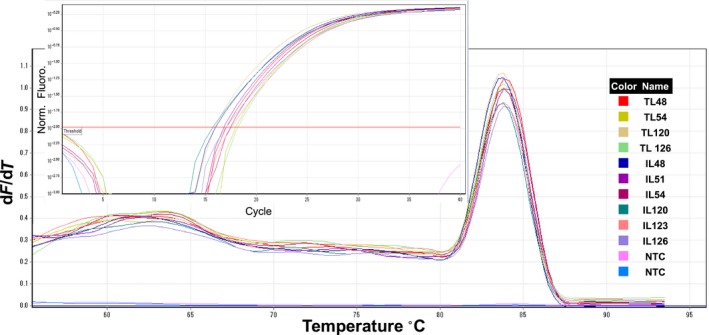
Amplification plots and melting curves for amplicons generated using cDNA from tissues that were taken from esparrago (ESP) genotype grown in pots and *in vitro*; qRT‐PCR analyses were performed using primers for 18S. M, 1 Kb ladder; biological repeat 1: TL48, buds and young leaves from pot samples grown at 15 °C; TL54, stems from potted plants grown at 15 °C; biological repeat 2; TL120, buds and young leaves from potted plants grown at 30 °C; TL126, stems from potted plants grown at 30 °C; IL48, leaves from *in vitro* plants grown at 15 °C; IL51, stems from *in vitro* plants grown at 15 °C; IL54, roots from *in vitro* plants grown at 15 °C; IL120, leaves from *in vitro* plants grown at 30 °C; IL123, stems from *in vitro* plants grown at 30 °C; IL126, roots from *in vitro* plants grown at 30 °C; NTC, negative control, water template.

We also performed qRT‐PCR analyses of the 18S housekeeping gene using cDNA that was synthesized from RNA that were isolated from cassava leaves, buds, young leaves, stems, and roots. All samples were amplified with normal Ct values and standard deviations that allowed accurate determinations of expression levels, and all *R* ^2^ values were greater than 0.98. Moreover, Ct values between samples did not differ significantly, reflecting high purity of extracted RNA and almost equal concentrations of cDNA (Figs 5 and 6). In a previous study of turmeric RNA, Ct values varied between reactions with total RNA that was isolated using differing methods 10. In contrast, we observed a single melting curve in both qRT‐PCR experiments, further indicating the purity and specificity of our amplified PCR fragments. Moreover, in previous non‐template control experiments, the absence of detectable amplicons prior to 37 cycles indicated low genomic DNA contamination 10. In the present non‐template controls, amplicons became detectable only after 40 PCR cycles, indicating very low DNA contamination levels in our isolated RNA (Figs 5 and 6).

In conclusion, we optimized a comprehensive protocol for isolating high‐quality total RNA from cassava tissues and achieved high yields of high‐quality RNA. This protocol has cost–benefits over commercially available kits and protocols and is successful in cassava tissues, such as roots and stems, for which TRIZOL‐related protocols failed. The resulting mRNA were of sufficient quality for qRT‐PCR or RNA sequencing analyses, suggesting that the present RNA isolation procedure could be adopted for related species of *Manihot* spp., which contain high concentrations of contaminating secondary metabolites. These developments in the RNA extraction protocol are expected to facilitate gene expression studies in cassava molecular breeding programs, particularly because this protocol can be applied using basic chemicals and general laboratory consumables that are easily accessible by cassava researchers in Africa, Asia, and South America and also because it provides a good balance of cost and efficiency. Furthermore, protocol will allow scientists in these geographies to produce very high‐quality and quantity RNA that can be used in studies that improve the reliability of cassava crops, which are an important source of nutrition.

## Conflict of interest

The authors declare no conflict of interest.

## Author contributions

BB and AB‐C contributed equally to this work, conducting experiments, collecting and analyzing data, as well as writing the article. OFC‐M helped in conducting parts of the experiments. HT and MI provided critical advice and comments on this research. LABL‐L supervised this research.

## Supporting information


**Fig. S1.** Conventional PCR using cDNA from total RNA.
**Fig. S2.** Conventional PCR using primers for the housekeeping gene *G3pdh* cDNAs corresponding to total RNA from various tissues.
**Fig. S3.** Electrophoresis run summary using Bioanalyzer 2100 expert_Plant RNA Nano shows RNAs in gels and electropherograms.
**Fig. S4.** Electropherograms of total RNA from cassava obtained using our method showing 18S and 25S rRNA regions with RNA concentrations and RIN values.
**Fig. S5.** Electrophoresis run summary using Bioanalyzer 2100 expert_High Sensitivity DNA Assays shows cDNA libraries in gels and electropherograms.
**Table S1.** Yields according to A260:A280 and A260:A230 ratios of isolated total RNA from cassava leaf tissues (HMC‐1 cultivar), which extracted by Trizol Method.
**Table S2.** Yields according to *A*_260_ : *A*_280_ and *A*_260_ : *A*_230_ ratios of isolated total RNA from various cassava tissues.
**Table S3**. The cost of extracted RNA based on our methodology and different RNA extraction kits.

## References

[1] Adeyemo OS , Chavarriaga P , Tohme J , Fregene M , Davis SJ and Setter TL (2017) Overexpression of Arabidopsis *FLOWERING LOCUS T* (*FT*) gene improves floral development in cassava (*Manihot esculenta*, Crantz). PLoS One 12, e0181460. 28753668 10.1371/journal.pone.0181460 PMC5533431

[2] Amuge T , Berger DK , Katari MS , Myburg AA , Goldman SL and Ferguson ME (2017) A time series transcriptome analysis of cassava (*Manihot esculenta* Crantz) varieties challenged with Ugandan cassava brown streak virus. Sci Rep 6, 32006. 10.1038/s41598-017-09617-z PMC5575035 28852026

[3] An D , Yang J and Zhang P (2012) Transcriptome profiling of low temperature‐ treated cassava apical shoots showed dynamic responses of tropical plant to cold stress. BMC Genom 13, 1–24. 10.1186/1471-2164-13-64 PMC3339519 22321773

[4] Li S , Yu X , Cheng Z , Yu X , Ruan M , Li W and Peng M (2017) Global gene expression analysis reveals crosstalk between response mechanisms to cold and drought stresses in cassava seedlings. Front Plant Sci 18, 1259. 10.3389/fpls.2017.01259 PMC5513928 28769962

[5] Liao W , Yang Y , Li Y , Wang G and Peng M (2016) Genome‐wide identification of cassava R2R3 MYB family genes related to abscission zone separation after environmental‐ stress‐induced abscission. Sci Rep 6, 32006. 10.1038/srep32006 PMC5004182 27573926

[6] Maruthi MN , Bouvaine S , Tufan HA , Mohammed IU and Hillocks RJ (2014) Transcriptional response of virus‐infected cassava and identification of putative sources of resistance for cassava brown streak disease. PLoS One 9, e96642. 24846209 10.1371/journal.pone.0096642 PMC4028184

[7] Utsumi Y , Tanaka M , Morosawa T , Kurotani A , Yoshida T , Mochida K , Matsui A , Umemura Y , Ishitani M , Shinozaki K *et al* (2012) Transcriptome analysis using a high‐density oligomicroarray under drought stress in various genotypes of cassava: an important tropical crop. DNA Res 19, 335–345. 22619309 10.1093/dnares/dss016 PMC3415295

[8] Xu J , Aileni M , Abbagani S and Zhang P (2010) A reliable and efficient method for total RNA isolation from various members of spurge family (*Euphorbiaceae)* . Phytochem Anal 21, 395–398. 20135710 10.1002/pca.1205

[9] Chang S , Puryear J and Cairney J (1993) A simple and efficient method for isolating RNA from pine trees. Plant Mol Biol Rep 11, 113–116.

[10] Deepa K , Sheeja TE , Santhi E , Sasikumar B , Cyriac A , Deepesh PV and Prasath D (2014) A simple and efficient protocol for isolation of high quality functional RNA from different tissues of turmeric (*Curcuma longa* L.). Physiol Mol Biol Plants 20, 263–271. 24757331 10.1007/s12298-013-0218-y PMC3988332

[11] Gehrig HH , Winter K , Cushman J , Borlan A and Taybi T (2000) An improved RNA isolation method for succulent plant species rich in polyphenols and polysaccharides. Plant Mol Biol Rep 18, 369–376.

[12] Hou P , Xie Z , Zhang L , Song Z , Mi J , He Y and Li Y (2011) Comparison of three different methods for total RNA extraction from Fritillaria unibracteata: a rare Chinese medicinal plant. J Med Plants Res 5, 2834–2838.

[13] Takahashi H , Kamakura H , Shiono K , Abiko T , Tsutsumi N , Nishizawa NK and Nakazono M (2010) A method for obtaining high quality RNA from paraffin sections of plant tissues by laser microdissection. J Plant Res 123, 807–813. 20221666 10.1007/s10265-010-0319-4

[14] Thanh T , Omar H , Abdullah MP , Chi VTQ , Noroozi M , Ky H and Napis S (2009) Rapid and effective method of RNA isolation from green microalga Ankistrodesmus convolutes. Mol Biotechnol 43, 148–153. 19507070 10.1007/s12033-009-9182-8

[15] Chomczynski P and Sacchi N (1987) Single‐step method of RNA isolation by acid guani‐diniumthiocyanate‐phenol‐chloroform extraction. Anal Biochem 162, 156–159. 2440339 10.1006/abio.1987.9999

[16] Gareth P , Asuncion LL , Marta V and Ester S (2006) Simple and rapid RNA extraction from freeze‐dried tissue of brown algae and seagrasses. Eur J Phycol 41, 97–104.

[17] Wang XC , Tain WM and Li YX (2008) Development of an efficient protocol of RNA isolation from recalcitrant tree tissues. Mol Biotechnol 38, 57–64. 18095190 10.1007/s12033-007-0073-6

[18] Wang L , Si Y , Dedow LK , Shao Y , Liu P and Brutnell TP (2011) A low‐cost library construction protocol and data analysis pipeline for Illumina‐based strand‐specific multiplex RNA‐seq. PLoS One 6, e26426. 22039485 10.1371/journal.pone.0026426 PMC3198403

[19] Castañeda‐Méndez O , Ogawa S , Medina A , Chavarriaga P and Gomez Selvaraj M (2017) A simple hydroponic hardening system and the effect of nitrogen source on the acclimation of *in vitro* cassava (*Manihot esculenta* Crantz). Vitro Cell Dev Biol 53, 75–85.

[20] Seki M , Narusaka M , Ishida J , Nanjo T , Fujita M , Oono Y , Kamiya A , Nakajima M , Enju A , Sakurai T *et al* (2002) Monitoring the expression profiles of 7000 Arabidopsis genes under drought, cold and high‐salinity stresses using a full‐length cDNA microarray. Plant J 31, 279–292. 12164808 10.1046/j.1365-313x.2002.01359.x

[21] Loomis MD (1974) Overcoming problems of phenolics and quinones in the isolation of plant enzymes and organelles. Methods Enzymol 31, 528–544. 4418408 10.1016/0076-6879(74)31057-9

[22] Utsumi Y , Tanaka M , Kurotani A , Yoshida T , Mochida K , Matsui A , Ishitani M , Sraphet S , Whankaew S , Asvarak T *et al* (2016) Cassava *(Manihot esculenta)* transcriptome analysis in response to infection by the fungus *Colletotrichum gloeosporioides* using an oligonucleotide‐DNA microarray. J Plant Res 129, 711–726. 27138000 10.1007/s10265-016-0828-x

[23] Wilson MC , Mutka AM , Hummel AW , Berry J , Chauhan RD , Vijayaraghavan A , Taylor NJ , Voytas DF , Chitwood DH and Bart RS (2017) Gene expression analysis provides insight into the physiology of the important staple food crop cassava. New Phytol 213, 1632‐1641. 28116755 10.1111/nph.14443 PMC5516207

[24] Bowrin V and Sutton F (2016) Inversion induced Manihot esculenta stem tubers express key tuberization genes; Mec1, RZF, SuSy1 and PIN2. Plant Signal Behav 11, e1115167. 26785907 10.1080/15592324.2015.1115167 PMC4871658

[25] Chun J , Zheng YF and Wang SH (2008) A RNA isolation method suitable for a wider range of materials. Prog Biochem Biophys 35, 591–597.

[26] Ghawana S , Paul A , Kumar H , Kumar A , Singh H , Bhardwaj PK , Rani A , Singh RS , Raizada J , Singh K *et al* (2011) An RNA isolation system for plant tissues rich in secondary metabolites. BMC Res Notes 4, 1–5. 21443767 10.1186/1756-0500-4-85 PMC3079660

[27] Wang L and Stegemann JP (2010) Extraction of high quality RN7A from polysaccharide matrices using cetlytrimethylammonium bromide. Biomaterials 31, 1612–1618. 19962190 10.1016/j.biomaterials.2009.11.024 PMC2813910

[28] Perry RP and Kelley DE (1972) The production of ribosomal RNA from high molecular weight precursors. J Mol Biol 70, 265–279. 4672957 10.1016/0022-2836(72)90538-4

[29] Kiefer E , Heller W and Ernst D (2000) A simple and efficient protocol for isolation of functional RNA from plant tissues rich in secondary metabolites. Plant Mol Biol Rep 18, 33–39.

[30] Schroeder A , Mueller O , Stocker S , Salowsky R , Leiber M , Gassmann M , Lightfoot S , Menzel W , Granzow M and Ragg T (2006) The RIN: an RNA integrity number for assigning integrity values to RNA measurements. BMC Mol Biol 7, 3–16. 16448564 10.1186/1471-2199-7-3 PMC1413964

[31] Sánchez CR , Villacreses J , Blanc N , Espinoza L , Martínez C , Pastor G , Manque P , Undurraga SF and Polanco V (2016) High quality RNA extraction from Maqui berry for its application in next‐generation sequencing. Springer Plus 5, 1243. 27536526 10.1186/s40064-016-2906-x PMC4970997

[32] Chaudhary S , Chaudhary PS and Vaishnani TA (2016) Small RNA extraction using fractionation approach and library preparation for NGS platform. J Adv Res Biotech 1, 7.

