# Supplementary material for: An optimized isolation protocol yields high‐quality RNA from cassava tissues (Manihot esculenta Crantz)
Source: FEBS Open Bio. 2019 Feb 20;9(4):814–25. doi: 10.1002/2211-5463.12561 (PMC6443859; doi:10.1002/2211-5463.12561)
Supplement: Supplementary file 1 — Fig. S1. Conventional PCR using cDNA from total RNA. Fig. S2. Conventional PCR using primers for the housekeeping gene G3pdh cDNAs corresponding to total RNA from various tissues. Fig. S3. Electrophoresis run summary using Bioanalyzer 2100 expert_Plant RNA Nano shows RNAs in gels and electropherograms. Fig. S4. Electropherograms of total RNA from cassava obtained using our method showing 18S and 25S rRNA regions with RNA concentrations and RIN values. Fig. S5. Electrophoresis run summary using Bioanalyzer 2100 expert_High Sensitivity DNA Assays shows cDNA libraries in gels and electropherograms. Table S1. Yields according to A260:A280 and A260:A230 ratios of isolated total RNA from cassava leaf tissues (HMC‐1 cultivar), which extracted by Trizol Method. Table S2. Yields according to A 260 : A 280 and A 260 : A 230 ratios of isolated total RNA from various cassava tissues. Table S3. The cost of extracted RNA based on our methodology and different RNA extraction kits. [file FEB4-9-814-s001.pdf]

Supporting information

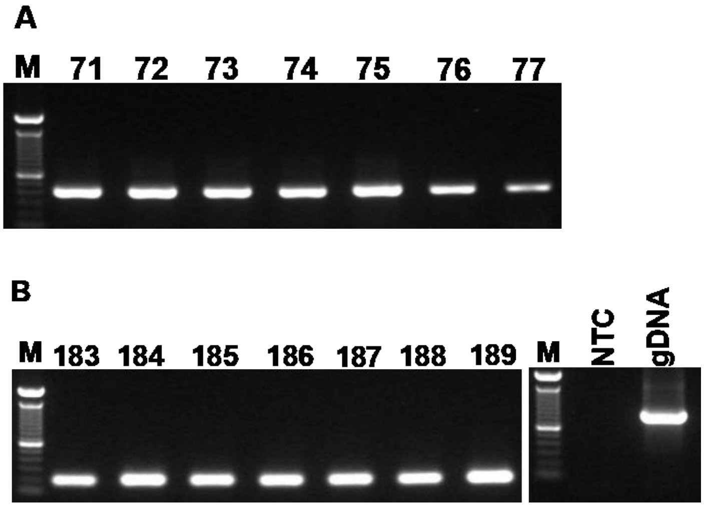

Fig. S1. Conventional PCR using cDNA from total RNA that was extracted from cassava HMC-1 (A) and ESP (B) samples grown in short day (SD) conditions for indicated times; analyses were performed using primers for *G3pdh*. M: 1 Kb ladder; 71, HMC-1, 0 h LD; 72, HMC-1 4 h LD; 73, HMC-1, 8 h LD; 74, HMC-1, 12 h LD; 75, HMC-1, 16 h LD; 76, HMC-1, 20 h LD; 77, HMC-1, 24 h LD; 183, ESP, 0 h LD; 184, ESP, 4 h LD; 185, ESP, 8 h LD; 186, ESP, 12 h LD; 187, ESP, 16 h LD; 188, ESP, 20 h LD; 189, ESP, 24 h LD; NTC, non-template negative control (water); gDNA, cassava genomic DNA.

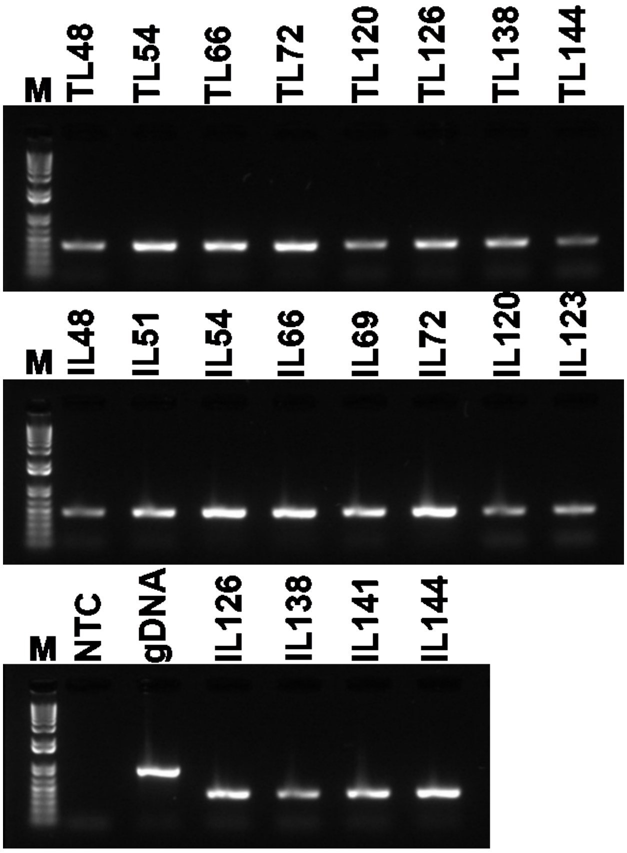

Fig. S2. Conventional PCR using primers for the housekeeping gene *G3pdh* cDNAs corresponding to total RNA from various tissues; cassava ESP genotype was grown in pots or *in vitro* at 15 °C and 30 °C under long day (LD) conditions from 16 h in growth chambers; M, 1 Kb ladder; Biological repeat 1: TL48, buds and young leaves from potted plants grown at 15 °C; TL54, stems from potted plants grown at 15 °C; Biological repeat 2: TL66, buds and young leaves from potted plants grown at 15 °C; TL72, stems from potted plants grown at 15 °C; Biological repeat 1: TL120, buds and young leaves from potted plants grown at 30 °C; TL126, stems from potted plants grown at 30 °C; Biological repeat 2: TL138, buds and young leaves from potted plants grown at 30 °C; IL141, stems from *in vitro* plants grown at 30 °C; TL144, stems from potted plants grown at 30 °C; Biological repeat 1: IL48, leaves from *in vitro* plants grown at 15 °C; IL51, stems from *in vitro* plants grown at 15 °C; IL54, roots *in vitro* plants grown at 15 °C; Biological repeat 2: IL66, leaves *in vitro* plants grown at 15 °C; IL69, stems *in vitro* plants grown at 15 °C; IL72, roots *in vitro* plants grown at 15 °C; Biological repeat 1: IL120, leaves from *in vitro* plants grown at 30 °C; IL123, stems from *in vitro* plants grown at 30 °C; IL126: roots from *in vitro* plants grown at 30 °C; Biological repeat 2: IL138; leaves from *in vitro* plants grown at 30 °C; IL141, stems from *in vitro* plants grown at 30 °C; IL144, roots from *in vitro* plants grown at 30 °C; NTC, negative control, water template; gDNA, cassava genomic DNA.

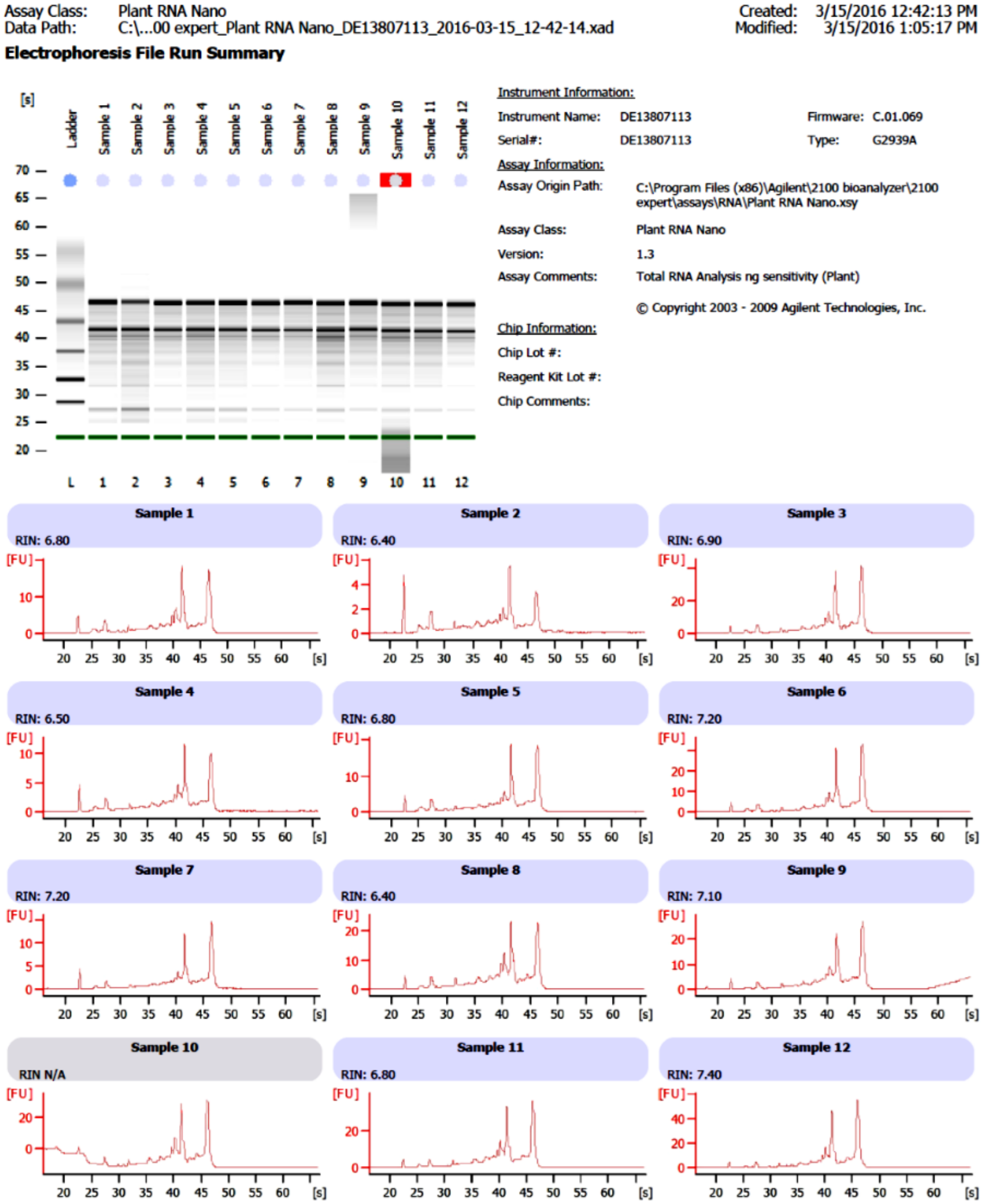

Fig. S3. Electrophoresis run summary using Bioanalyzer 2100 expert_Plant RNA Nano shows RNAs in gels and electropherograms; total RNAs were extracted from cassava leaves at indicated time points. The graph shows RNA quality according to ribosomal integrity numbers (RINs).

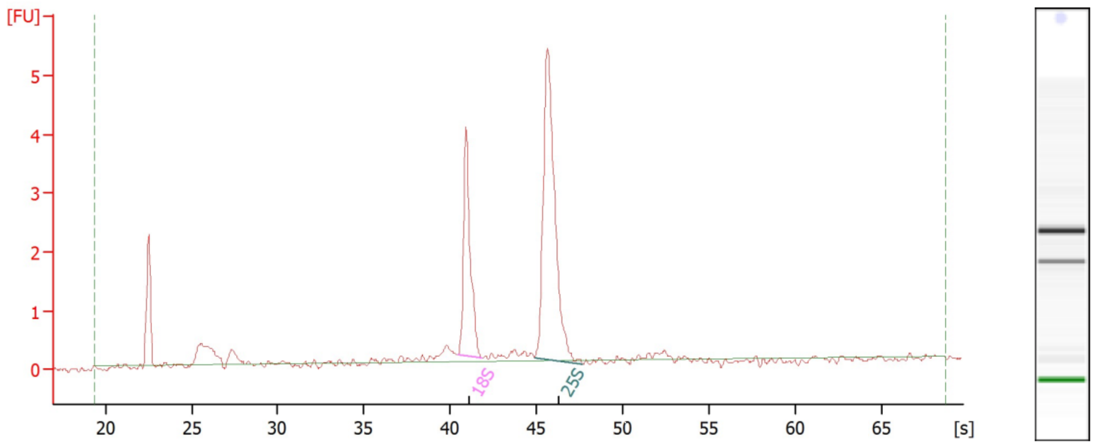

Cassava (Cultivar: HMC-1) fresh leaf from photoperiod experiment

- **RNA Concentration**: 21 ng/µl
- **rRNA Ratio** [25s / 18s]: 2.1
- **RNA Integrity Number (RIN)**: 9.6 (B.02.08)

Fig. S4. Electropherograms of total RNA from cassava obtained using our method showing 18S and 25S rRNA regions with RNA concentrations and RIN values. The RNA was from fresh leaves of Cassava HMC-1 cultivar. RNAs were visualized in denaturing agarose gels stained with SYBr safe. RNAs were analyzed using Agilent RNA 6000 Nano Assays in a 2100 Bioanalyzer (Agilent Technologies) and were then used for qRT-PCR.

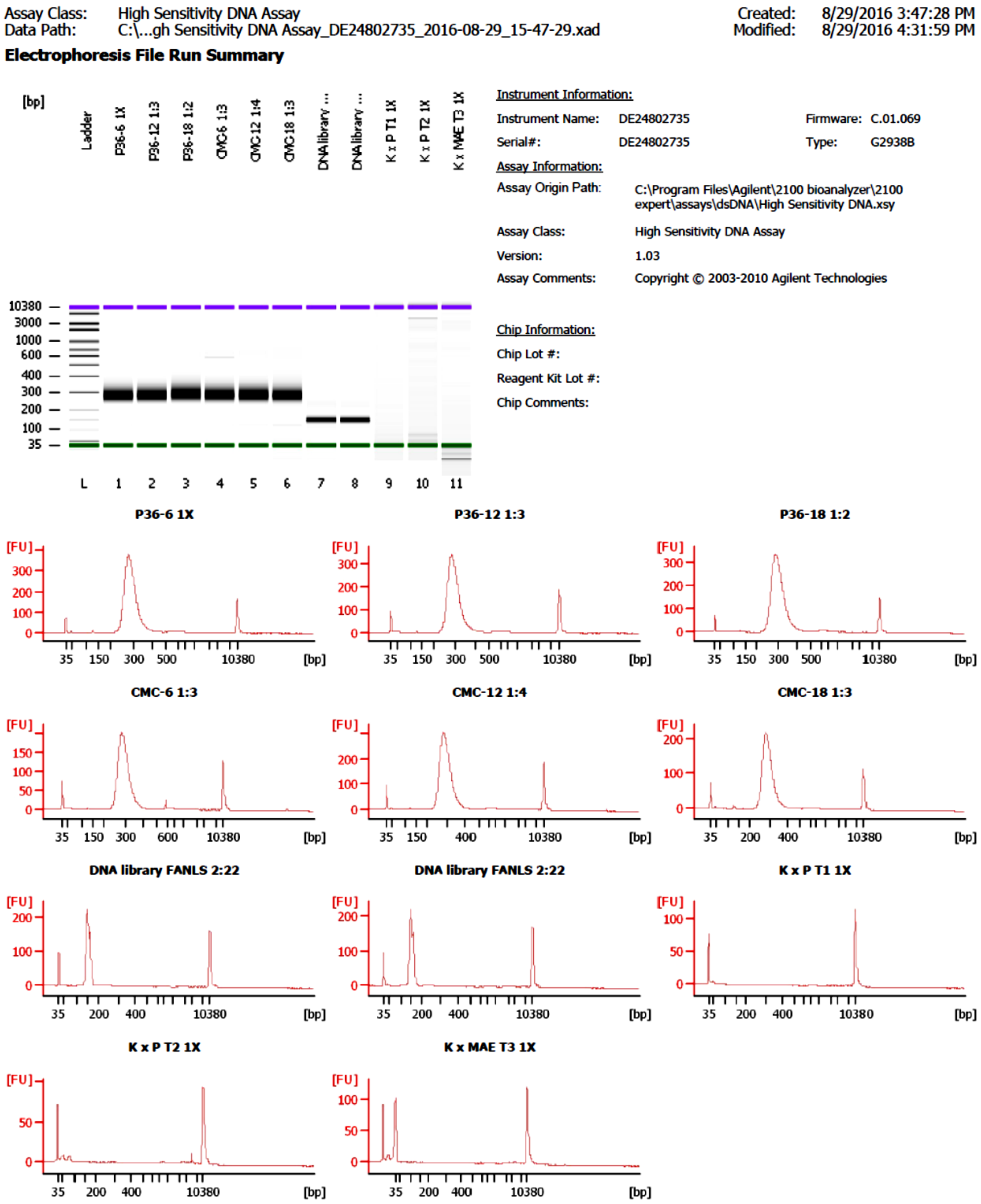

Fig. S5. Electrophoresis run summary using Bioanalyzer 2100 expert_High Sensitivity DNA Assays shows cDNA libraries in gels and electropherograms. The cDNA libraries were generated using total RNAs from cassava leaves. The graph shows length distribution curves of sequencing libraries that were generated using a low-cost library construction protocol [28].

Table S1. Yields according to A260:A280 and A260:A230 ratios of isolated total RNA from cassava leaf tissues (HMC-1 cultivar), which extracted by Trizol Method

sample	ng/ul	260/280	260/320
1	45.1	1.57	0.66
2	46.8	1.12	0.26
3	461.8	0.93	-6.26
4	-222.9	0.71	0.4
5	352..4	0.89	-8.37
6	83	1.08	0.29
7	73.7	1.72	0.86
8	132.2	1.75	0.91
9	375.5	1	1.73
10	121.7	1.81	0.97
3-1	211.1	0.85	0.63
3-2	57.8	1.28	0.45
3-3	63.2	1.44	0.55
4-1	325.4	1.3	0.41
4-2	1037.5	0.99	0.37
4-3	811.7	0.8	0.56

Table S2: Yields according to A_{260}:A_{280} and A_{260}:A_{230} ratios of isolated total RNA from various cassava tissues.

Name	Tissue	Yield (ng/µl)	260/ 280	260/ 230
71	HMC-1 0H-SD, Third leaf	2580.5	2.05	2.21
72	HMC-1 4H -SD, Third leaf	1729	2.05	2.24
73	HMC-1 8H-SD, Third leaf	2876	2.02	2.19
74	HMC-1 12H -SD, Third leaf	2121.8	2.06	2.07
75	HMC-1 16H -SD, Third leaf	1916.5	2.06	2.26
76	HMC-1 20H-SD, Third leaf	1270	2.14	2.03
77	HMC-1 24H-SD, Third leaf	3132.9	2.08	2.19
183	Esparrago 0H-SD, Third leaf	1701.8	2.19	2.34
184	Esparrago 4H -SD , Third leaf	2232.7	2.15	2.18
185	Esparrago 8H-SD, Third leaf	2820.3	2.1	2.18
186	Esparrago 12H -SD, Third leaf	2582.6	2.07	2.18
187	Esparrago 16H -SD, Third leaf	2133.2	2.08	2.29
188	Esparrago 20H-SD , Third leaf	1800.5	2.1	2.24
189	Esparrago 24H-SD , Third leaf	2045.5	2.09	2.25
TL66	HMC-1Buds and young leaves Rep II (from Pot in 15◦C)	1826.3	2.11	2.13
TL72	HMC-1 Stem Rep II (from Pot in 15◦C)	1115	2.09	2.05
TL120	HMC-1Buds and young leaves Rep I (from Pot in 30◦C)	1992.6	2.08	2.03
TL126	HMC-1 Stem Rep I (from Pot in 30◦C)	470.1	2.06	2.04
TL138	HMC-1Buds and young leaves Rep(from Pot in 30◦C)	1943.6	2.09	2.01

Table S2:*Continue…*

Name	Tissue	Yield (ng/µl)	260/280	260/230
TL144	HMC-1 Stem Rep II (from Pot in 30◦C)	1338.9	2.11	2.04
IL48	HMC-1 Leaves RepI (from *In-vitro* samples in 15◦C)	2217.5	2.02	1.86
IL51	HMC-1 Stem from RepI (from *In-vitro* samples in 15◦C)	1217.3	2.12	2.13
IL54	HMC-1 Roots from RepI (from *In-vitro* samples in 15◦C)	1561.3	2.11	2.07
IL66	HMC-1 Leaves RepII (from *In-vitro* samples in 15◦C)	1343.3	2.12	2.11
IL69	HMC-1 Stem from RepII (from *In-vitro* samples in 15◦C)	213.1	2.1	2.06
IL72	HMC-1 Roots from RepII (from *In-vitro* samples in 15◦C)	753.4	2.14	2.22
IL120	HMC-1 Leaves RepI (from *In-vitro* samples in 30◦C)	2188.9	2.07	2.21
IL123	HMC-1 Stem from RepI (from *In-vitro* samples in 30◦C)	783.7	2.11	2.19
IL144	HMC-1 Roots from RepII (from *In-vitro* samples in 30◦C)	605.8	2.08	1.81

Table S3: The cost of extracted RNA based on our methodology and different RNA extraction kits.

Methology	Description	Unit	QTY	Price* (USD/$)	QTY/ sample	Price per sample (USD)	RNA yield
Our methodology**	Eppendorf Safe-Lock Tubes™, 2.0 ml, per 500 pieces, Cat #022363352	PCK	1	54.83	1 tube x 6	0.65196	
	Acid Phenol:Chloroform, pH 4.5 (with IAA, 125:24:1) Ambion	BT X 400 mL	1	180	1 sample (0.5 mL)	0.108	
	Lithium chloride solution 8M, REF L1026, SIGMA	BT X 500 mL	1	88.9	1 sample (0.5 mL)	0.03556	
	Sodium chloride solution 5M, S5150-1L SIGMA	BT X 1 L	1	65	1 sample (0.05 mL)	0.0013	
	Ultrapure 1M Tris-HCl pH: 7.5, 15561-021 SIGMA	BT X 1 L	1	44.16	1 sample (0.25 mL)	0.004416	
	0.5M EDTA, pH 8.0 Molecular Biology Grade, Promega	BT X 400 mL	1	150	1 sample (0.125 mL)	0.1875	
	2-Mercaptoethanol M6250, SIGMA	BT X 500 mL	1	19.01	1 sample (0.05 mL)	0.0031604	
	SDS Sodium Dodecyl sulfate, L3771 SIGMA	BT X 1 Kg	1	415	1 sample (0.025 g)	0.00415	
	POLYVINYLPYRROLIDONE AVERAGE MOL WT 40,000 -PVP40 SIGMA	BT X 1 Kg	1	329	1 sample (0.05 g)	0.00658	
	Absolute Alcohol, Merck	BT X 2.5 L	1	64.42	1 sample (2 mL)	0.051536	
	Chloroform, Merck	BT X 2.5 L	1	182.99	1 sample (4 mL)	0.292784	
	Isoamyl Alcohol, Merck	BT X 2.5 L	1	42.84	1 sample (10 ul)	0.0001712	
	UltraPure Water, Invitrogen	PK 10 X 500 mL	1	234	1 sample (1 mL)	0.03144	
						1.3906176	6 ug
						0.2317696	1 ug
QIAGEN KIT	Qiagen Plant RNeasy, 50 samples,	KT X 50 RXN	1	319	1 sample	1.59	
	Absolute Alcohol Merck***	BT X 2.5 L	1	31	1 sample (2 mL)	0.062	
						7.642	1 ug
						45.852	6 ug
Spectrum Plant Total RNA kit	Spectrum Plant Total RNA kit, SIGMA, STRN50-1KT, 50 samples	KT X 50 RXN	1	288	1 sample	5.16	
	Absolute Alcohol Merck***	BT X 2.5 L	1	31	1 sample (2 mL)	0.062	
	Eppendorf Safe-Lock Tubes™, 2.0 ml, per 500 pieces Cat #022363352***	PCK	1	54.83	1 tube	0.10966	
						0.98861	1 ug****
						5.93166	6 ug****
PicoPure RNA Isolation Kit	PicoPure RNA Isolation Kit 200 RXN, Life Technologies	KT X 200 RXN	1	1598	1 sample	4.194	6 ug
						0.4794	1 ug

*All prices are from the catalog, including those of the kit

**Calculated prices for 1 sample / 1 mL of buffer, from 100 mg of tissue

***Reagents are not included in the kit

****yields can vary depending on the age, health and stress level of the plant (Manufacturer's note)
